# Risk Factors Associated With Methicillin‐Resistant *Staphylococcus aureus* Infections in Patients With Diabetic Foot Ulcers in Tehran

**DOI:** 10.1155/cjid/5695498

**Published:** 2026-04-12

**Authors:** Haniyeh Khalili, Mona Mahrooghi, Shahin Najar-Peerayeh, Shabnam Zeighamy Alamdary, Hossein Najd Sepas, Bita Bakhshi

**Affiliations:** ^1^ Faculty of Medical Sciences, Department of Bacteriology, Tarbiat Modares University, Tehran, Iran, modares.ac.ir; ^2^ Department of Microbiology, School of Medicine, Tehran University of Medical Sciences, Tehran, Iran, tums.ac.ir; ^3^ Department of General Surgery, School of Medicine, Iran University of Medical Sciences, Tehran, Iran, iums.ac.ir

**Keywords:** diabetic foot ulcers, MRSA, MSSA, *Staphylococcus aureus*

## Abstract

**Background:**

Foot ulcer is a common complication of diabetes mellitus, which is associated with high morbidity and mortality rates among diabetic patients. This study aimed to identify risk factors associated with methicillin‐resistant *Staphylococcus aureus* (*S. aureus*) (MRSA) infections and their epidemiology among patients with diabetic foot ulcers (DFUs).

**Methods:**

A total of 211 wound samples were collected from patients with DFUs in five educational hospitals in Tehran during 2017–2019. The presence of certain virulence markers (α‐haemolysin (*hlα*), phenol‐soluble modulins (*psmα*), accessory gene regulator (*agr*), exfoliative toxin A, B (*eta etb*) and toxic shock syndrome toxin‐1 (*tsst-1*)) was investigated by polymerase chain reaction (PCR), and a multiplex PCR assay was used to screen for staphylococcal cassette chromosome (SCC*mec*) types. Antibiotic susceptibility testing (AST) was performed by the Kirby–Bauer disk diffusion method.

**Results:**

A significant proportion of patients with DFUs had Type 2 diabetes. Grading of DFUs revealed that Grade 4 was the most common grade among patients. The majority of patients were infected with *S. aureus* (45.3%) and *Escherichia coli* (*E. coli*) (29.4%). A total of 59 *S. aureus* isolates were identified, of which 37 (62.7%) were MRSA, and 22 (37.2%) were methicillin‐susceptible *S. aureus* (MSSA). MRSA isolates were mostly positive for the *hlα* (57.6%) and *psmα* (50.8%) genes. The *tsst-*1 gene was detected in 8.5% of MRSA strains, while the *eta* (3.4%) and *etb* (5.1%) genes had the lowest prevalence in MRSA isolates. According to the results, SCC*mec* Type III (48.6%) was the most common subtype among MRSA isolates, followed by SCC*mec* II (16.2%). The antimicrobial susceptibility testing results revealed that linezolid (66.1%), gentamycin (66.1%) and mupirocin (62.7%) were the most effective antibiotics against MRSA isolates, respectively. In contrast, MRSA strains showed the highest resistance to cefoxitin and penicillin. Finally, the risk factors in patients with DFU in this study were diastolic hypertension, cardiovascular diseases, renal failure, neuropathy disease and hospitalization history.

**Conclusions:**

The findings underscore the critical importance of a comprehensive understanding of patients’ medical records and underlying conditions. In addition, accurate assessment of microbial prevalence and antibiotic susceptibility is essential for the effective management of DFUs.

## 1. Introduction

Diabetes mellitus is a prevalent endocrine and metabolic syndrome that is considered a significant global public health challenge. Epidemiological studies have revealed that approximately 150 million people worldwide suffer from diabetes, and this number is projected to increase to more than 300 million by 2025 and 783 million by 2045 [1]. According to recent studies, it is estimated that approximately 200 million people in low‐ and middle‐income countries (LMIC) suffer from diabetes [2]. The prevalence of this condition in Iran varies from 3% to 17% in different geographical areas [3]. The projected prevalence of diabetes and prediabetes among the Iranian population aged 18 years and above in 2021 was estimated to be approximately 14.15% and 24.79%, respectively [4]. This represents a substantial increase of 45.5% in diabetes prevalence compared to 2016 [4]. According to the latest national survey of noncommunicable disease (NCD) risk factors, 15.14% of the Iranian population over 25 years of age had diabetes in 2021 [5].

The number of people with diabetes in Iran is expected to rise to 9.2 million by 2030 if effective preventive measures are not implemented [6]. The most common type of diabetes is Type 2 diabetes, which accounts for about 80%–85% of all diabetic cases [7]. The clinical manifestations of diabetes mellitus are diverse and could vary significantly from patient to patient. These manifestations may include retinal, renal, and peripheral neuropathy, as well as peripheral arterial disease, which could result in foot injuries and ulcers [8]. Among all diabetes complications, diabetic foot ulcer (DFU) is the most common and serious complication, affecting approximately 15% of diabetic patients [9].

Bacterial infections are one of the most common causes of DFUs, which could lead to severe complications such as cellulitis, sepsis, gangrene, and limb amputation if left untreated [10]. In fact, DFUs are the leading cause of hospitalization among diabetic patients [11]. Damaged skin is primarily affected by bacterial colonization, which could expose deep tissues to a wide variety of bacteria [12, 13]. Deep and chronic wounds are often colonized by aerobic Gram‐negative or obligate anaerobic bacteria [14, 15]. Mild bacterial infections are typically caused by a single bacterial strain, while severe infections are often associated with more than one strain [12]. DFUs are classified into six different grades based on the severity and extent of the wound, and the first four grades are based on the physical features of the wound and the soft tissues of the foot [16, 17]. Grade 0 indicates the presence of foot symptoms such as pain, while Grade 1 refers to a superficial ulcer [18]. Grade 2 denotes a deep ulcer that extends to the bone or joint, and Grade 3 describes deep ulcers with abscess or osteomyelitis [19]. The last two grades, Grades 4 and 5, are distinct because they are based on the extent of gangrene and loss of perfusion in the foot. Grade 4 denotes partial gangrene of the foot, while Grade 5 refers to complete gangrene of the foot [20].

DFU is a commonly diagnosed clinical manifestation of diabetes. Although microbiological diagnosis has limitations, it is still recommended due to the high prevalence of microorganisms in chronic wounds [21]. The management of DFUs is complicated by the emergence of antibiotic resistance, which could result in treatment failure due to the high prevalence of multi–drug‐resistant (MDR) bacteria [22]. This is a serious threat to patients’ lives and could prolong the duration of treatment [23]. Studies have shown that *S. aureus*, a Gram‐positive coccus, is the most common pathogen isolated from DFUs [24, 25].

Early diagnosis and timely treatment of infected patients could significantly reduce the bacterial load of *S. aureus* at the site of infection and improve the clinical features of DFUs [26]. However, an important issue in recent years has been the isolation of multidrug‐resistant organisms (MDROs) from diabetic foot infections (DFIs), owing to repeated hospitalizations, frequent exposure to antibiotic therapy, and low antibiotic concentrations in infected tissues due to poor arterial supply [27]. Previous research has indicated that methicillin‐resistant *Staphylococcus aureus* (MRSA) is the most prevalent resistant microorganism in lesions, accounting for over one‐third of isolates [28, 29].

Identification of vancomycin‐resistant *S. aureus* (VRSA) strains in DFIs has posed a significant challenge in the treatment process [30, 31]. DFIs caused by MRSA exhibit worse outcomes compared to those caused by methicillin‐susceptible S. aureus (MSSA) or other pathogens [32]. Several studies have indicated that patients suffering from various types of MRSA infections are five times more at risk of in‐hospital death compared to those without MRSA infection [33–35]. The current research aimed to identify MRSA and MSSA strains and other bacteria associated with foot ulcers in patients with Type II diabetes mellitus. Additionally, the study also investigated the prevalence of virulence gene markers (*hlα*, *psmα*, *agr*, *eta*, *etb* and *tsst-1*) and staphylococcal cassette chromosome *mec* (SCC*mec*) types in MRSA strains.

## 2. Materials and Methods

### 2.1. Sample Collection

A total of 211 wound samples were collected from patients who suffered from foot ulcers, had a history of hospitalization in the past one year and were admitted to five teaching hospitals in Tehran from 2017 to 2019. The status and grade of DFUs were assessed by a physician. The DFUs included in this study were classified according to the Wagner classification system [36]. This widely used system categorizes foot ulcers based on depth, presence of infection and gangrene into six grades (0–5). Samples were collected from ulcers ranging from superficial wounds (Grade 1) to those with deep infection and gangrene (Grades 3–5) [36]. This classification helped in stratifying the samples according to severity, ensuring appropriate analysis of microbiological and clinical characteristics. Wound samples were collected by scraping the wound base and collecting debris through wound base swabbing, needle aspiration or tissue biopsy. The collected samples were promptly placed into thioglycolate broth medium and immediately sent to the Bacteriology Department of Tarbiat Modares University.

### 2.2. Microbiological Examinations

The collected specimens were cultured on blood agar medium and incubated at 37°C for 24 h. The Gram staining method was used for the initial identification of bacterial samples. Gram‐negative bacilli were characterized using *Enterobacteriaceae* tests [37], and Gram‐positive cocci were characterized by mannitol salt agar (MSA), tube coagulase and deoxyribonuclease (DNase) tests for further phenotypic analysis [38–40]. The initial samples were grown on MSA media (Merck, Darmstadt, Germany) to distinguish members of the *Staphylococcus* genus because they could tolerate 7.5% salt concentrations. The samples obtained from MSA plates were subjected to the tube coagulase test. This test is based on the ability of *S. aureus* to clot blood plasma owing to its catalase‐positive nature. Then, the DNase test was performed on DNase media (Merck, Darmstadt, Germany) to distinguish *S. aureus* isolates. DNase‐positive isolates were characterized by clear zones surrounding the inoculation band, while the medium away from the inoculation band appeared opaque and whitish [41]. In addition, all *S. aureus* strains were tested for haemolysin *α*, *β* and *γ*. They were cultured on blood agar and placed in an incubator at 37°C for 24 h, and then, their ability to produce haemolysin was checked.

Antibiotic susceptibility testing (AST) was performed on *S. aureus* isolates using the Kirby–Bauer disk diffusion method [42] on Müeller–Hinton agar (MHA) (Merck, Darmstadt, Germany) plates according to CLSI guidelines (Clinical and Laboratory Standards Institute, 2017) [43]. The antibiotic disks used were as follows: cefoxitin (30 μg), gentamicin (10 μg), erythromycin (15 μg), tetracycline (30 μg), rifampin (5 μg), ciprofloxacin (5 μg), trimethoprim–sulfamethoxazole (25 μg), linezolid (30 μg), mupirocin (200 μg), chloramphenicol (30 μg), clindamycin (2 μg), amikacin (30 μg) and penicillin (10 μg) (MAST company). *S. aureus* ATCC 25923 strain was used as a control [44].

### 2.3. Genomic DNA Preparation and *nuc* Gene Detection

Genomic DNA of Gram‐positive bacteria was extracted using an extraction kit (Gene Transfer Pishgaman, Iran) as per the manufacturer’s protocol. The *nuc* gene was utilized for genetic confirmation of isolates that showed negative results in phenotypic tests. The extracted DNA samples were subjected to electrophoresis on 0.8% (w/v) agarose gels to assess their quality and then stored at −20°C until further use for polymerase chain reaction (PCR) analysis. PCR was carried out to identify the presence of the *nuc* gene in all strains using specific primers (*nuc*‐F GCG​ATT​GAT​GGT​GAT​ACG​GTT and *nuc*‐R AGC​CAA​GCC​TTG​ACG​AAC​TAA​AGC), which yielded DNA fragments of 278 bp.

### 2.4. Detection of MRSA Strains and SCC*mec* Typing

The present study employed PCR analysis to confirm the presence of the *mec*A gene in all strains using specific primers (F: GTG​AAG​ATA​TAC​CAA​GTG​ATT and R: ATG​CGC​TAT​AGA​TTG​AAA​GGA), which yielded 146‐bp fragments. Furthermore, MRSA isolates detected among all *S. aureus* strains were screened for cefoxitin resistance (30 μg). Additionally, a multiplex PCR‐based method was carried out for SCC*mec* typing. The oligonucleotide primers employed to amplify the genes of interest are listed in Table 1.

**TABLE 1:** Oligonucleotide sequences and amplicon size of virulence genes.

Primer	Sequence (5ʹ ⟶ 3ʹ)	Size (bp)
SCC*mec I*	F: GCT​TTA​AAG​AGT​GTC​GTT​ACA​GG	613
	R: GTT​CTC​TCA​TAG​TAT​GAC​GTC​C	

SCC*mec II*	F: CGT​TGA​AGA​TGA​TGA​AGC​G	398
	R: CGA​AAT​CAA​TGG​TTA​ATG​GAC​C	

SCC*mec III*	F: CCA​TAT​TGT​GTA​CGA​TGC​G	280
	R: CCT​TAG​TTG​TCG​TAA​CAG​ATC​G	

SCC*mec Iva*	F: GCC​TTA​TTC​GAA​GAA​ACC​G	776
	R: CTA​CTC​TTC​TGA​AAA​GCG​TCG	

SCC*mec IVb*	F: TCT​GGA​ATT​ACT​TCA​GCT​GC	493
	R: AAA​CAA​TAT​TGC​TCT​CCC​TC	

SCC*mec IVc*	F: ACA​ATA​TTT​GTA​TTA​TCG​GAG​AGC	200
	R: TTG​GTA​TGA​GGT​ATT​GCT​GG	

SCC*mec IVd*	F: CTC​AAA​ATA​CGG​ACC​CCA​ATA​CA	881
	R: TGC​TCC​AGT​AAT​TGC​TAA​AG	

SCC*mec V*	F: GAA​CAT​TGT​TAC​TTA​AAT​GAG​CG	325
	R: TGA​AAG​TTG​TAC​CCT​TGA​CAC​C	

*Psmα*	F: TAT​CAA​AAG​CTT​AAT​CGA​ACA​ATT​C	176
	R: CCC​CTT​CAA​ATA​AGA​TGT​TCA​TAT​C	

*Eta*	F: CTA​GTG​CAT​TTG​TTA​TTC​AA	119
	R: TGC​ATT​GAC​ACC​ATA​GTA​CT	

*Etb*	F:ACGGCTATATACATTCAATT	200
	R:TCCATCGATAATATACCTAA	

*tsst-1*	F: TTA​TCG​TAA​GCC​CTT​TGT​TG	398
	R: TAA​AGG​TAG​TTC​TAT​TGG​AGT​AGG	

*Hlα*	F: CGG​TAC​TAC​AGA​TAT​TGG​AAG​C	176
	R: TGG​TAA​TCA​TCA​CGA​ACT​CG	

*agr I-R*	GTC​ACA​AGT​ACT​ATA​AGC​TGC​GAT	440

*agr II-R*	GTA​TTA​CTA​ATT​GAA​AAG​TGC​CAT​AGC	572

*agr III-R*	CTG​TTG​AAA​AAG​TCA​ACT​AAA​AGC​TC	406

*agr IV-R*	CGATAATGCCGTAATAC CCG	588

### 2.5. *Agr* Genotyping by Duplex PCR

The *agr* Types I–IV were detected by duplex PCR assay [45]. The oligonucleotide primers used to amplify the desired genes are listed in Table 1. Moreover, molecular identification of genes like *tsst-*1, *hla*‐α, *psmα, eta* and *etb* was performed.

### 2.6. Biofilm Formation and Haemolysis Pattern

To investigate the binding and biofilm formation ability of *S. aureus* isolates, the microtitre plate method was done on BHI3V medium with 1% glucose in 96‐well tissue culture plates by applying a semiquantitative adherence test [46, 47]. The biofilm formation ability of the isolates was calculated based on the amount of crystal violet absorption into the cells in the biofilm, and the optical density of each well was determined at a wavelength of 570 nm. The results were analysed using a cut‐off. *S. aureus* ATCC 35556 was used as a positive control and a strong biofilm producer, and *S. epidermidis* ATCC 12228 was used as a negative control. This assay was carried out in triplicate for each strain. The cut‐off calculation method for each group, which was established previously [48], is given in Table 2.

**TABLE 2:** Calculation method of biofilm formation ability based on microtitre plate method.

Calculation of cut‐off	Ability to form biofilm
OD^1^ > 4 × ODc^2^	Strong
2 × ODc < OD ≤ 4 × ODc	Intermediate
ODc < OD ≤ 2 × ODc	Weak
OD ≤ ODc	No connection

^1^Optical density.

^2^ODc = average of OD negative control + (3 × SD of negative control).

### 2.7. Data Analysis

Data were reported as mean ± standard error of the mean (SEM). The statistical analyses were conducted using the chi‐square and Fisher’s exact test, with the *R* statistical software (v.4.4) [49]. The statistical significance was established when the *p* value was less than 0.05.

## 3. Results

### 3.1. Clinical Data and Microbiological Considerations

**FIGURE 1:**
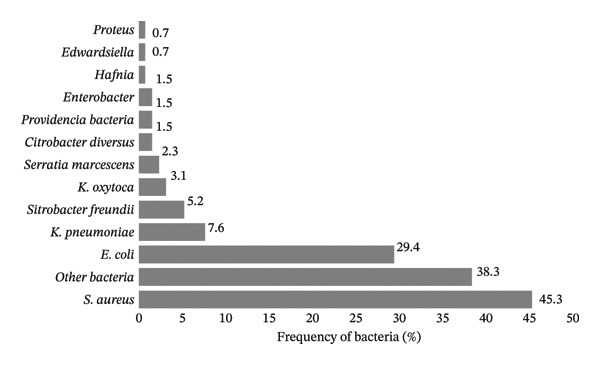
Frequency of bacteria in diabetic foot ulcer samples. The most and least prevalent bacteria were *S. aureus* and *Edwardsiella*, respectively.

A total of 211 patients with DFIs were hospitalized from 2017 to 2019 and studied in this research. Figure 1 shows the total number and type of bacteria isolated from DFUs of the studied patients (*n* = 211). Etiological evaluations showed that the most common bacterial isolates in DFUs were *S. aureus* (*n* = 59, 45.3%), *Escherichia coli* (*n* = 37, 29.4%) and *Klebsiella pneumoniae* (*n* = 10, 7%), respectively (Figure 1).

### 3.2. General Information of Patients With DFUs

**FIGURE 2:**
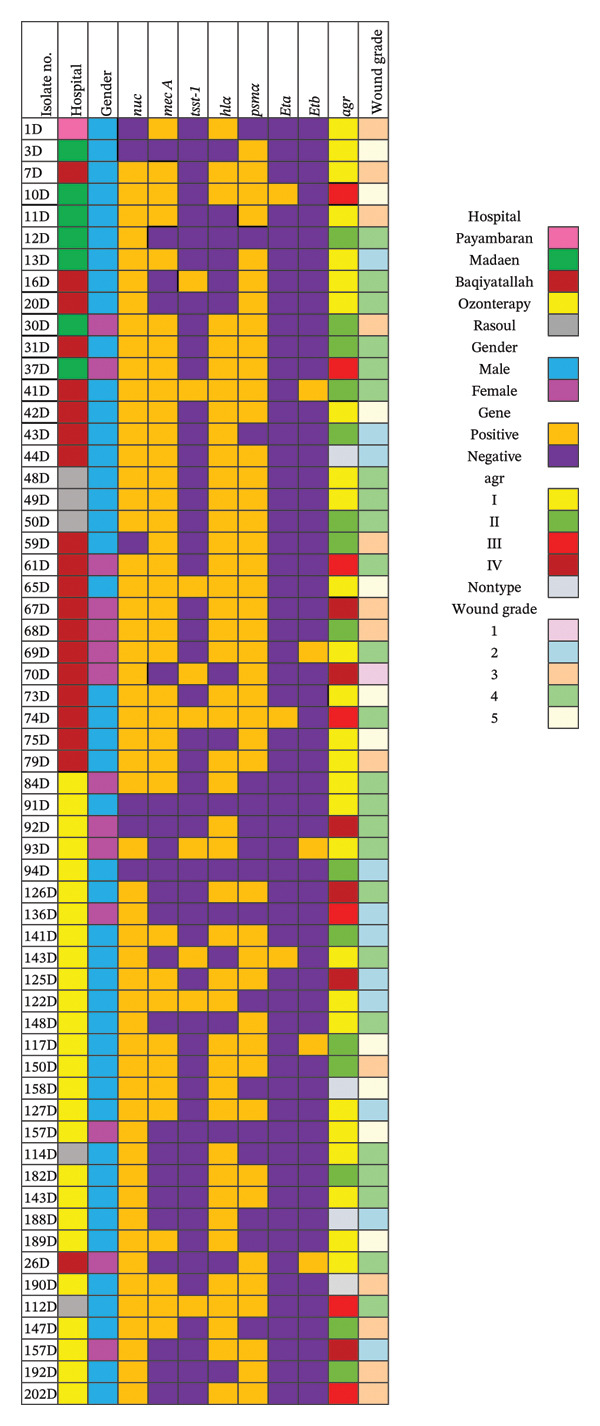
Heatmaps A&B show bacteriological aetiology of diabetic foot infections based on bacterial virulence factors, *agr* genotypes, antimicrobial resistance profiles and wound grades.

Heatmaps in Figure 2 depict the distribution of bacterial isolates obtained from DFUs in various hospitals. The findings revealed that *S. aureus* isolates were predominantly more prevalent among male patients (76%), while only 24% of the isolates were obtained from female patients. Out of 59 *S. aureus* isolates, 37 (62.7%) were MRSA, and 22 (37.3%) were MSSA. The prevalence of MSSA and MRSA isolates was equal in women (11.9%), while the prevalence of MRSA isolates in men was twice that of MSSA isolates (50.8% vs 25.4%) (Table 3). The average age of patients infected with MSSA and MRSA was 59 and 62 years, respectively (Table 3). The grading results of DFUs revealed that Grade 4 was the most prevalent grade among patients (*n* = 24), while only one sample belonged to Grade 1. Notably, more than 50% of Grade 4 foot ulcers were infected with MSSA, while wounds with other grades were more infected with MRSA. In total, 10 samples were classified as Grade 5, 24 samples as Grade 4, 13 samples as Grade 3, 11 samples as Grade 2, and 1 sample as Grade 1 (Tables 3 and 4).

**TABLE 3:** Demographic and clinical data of subjects with diabetic foot infections.

Variables	Data source	MSSA patients *N* (%)	MRSA patients *N* (%)	Enterobacteriaceae *N* (%)	Others *N* (%)	Statistical significance
*Sex*						
Female	Questionnaire	7 (31.81)	7 (18.91)	16 (22.53)	23 (28.39)	0.57
Male		15 (68.18)	30 (81.08)	55 (77.46)	58 (71.60)	

*Diabetic foot ulcers grading*						
1	Physician	1 (4.54)	0 (0.0)	8 (11.26)	7 (8.64)	**< 0.001**
2		4 (18.18)	7 (18.91)	10 (14.08)	10 (12.34)	
3		2 (9.09)	11 (29.72)	13 (18.30)	15 (18.51)	
4		13 (59.09)	11 (29.72)	18 (25.35)	48 (59.25)	
5		2 (9.09)	8 (21.62)	22 (30.98)	1 (1.23)	

*Underlying diseases*						
Hypertension	Physician and medical records	4 (18.18)	13 (35.13)	22	22	**0.002**
Cardiovascular diseases		6 (27.27)	9 (24.32)	14	27	**< 0.001**
Renal failure		4 (18.18)	3 (8.10)	0	15	**< 0.001**
Neuropathy		0	0	6	14	**< 0.001**
Nephropathy		1 (4.54)	8 (21.62)	17	10	**0.002**

*History of admission*						
Yes	Questionnaire	14 (63.63)	27 (72.97)	41 (57.74)	47 (58.02)	0.45
No		8 (36.36)	10 (27.02)	30 (42.25)	34 (41.97)	

*Association with skin diseases*						
Cellulitis	Physician and medical records	3 (13.63)	10 (27.02)	20 (28.16)	32 (39.50)	**< 0.001**
Deep soft tissue involvement		17 (77.27)	20 (54.05)	47 (66.19)	31 (38.27)	**< 0.001**
Bone involvement		4 (18.18)	4 (10.81)	10 (14.08)	19 (23.45)	**< 0.001**
Topical necrosis		5 (22.72)	11 (29.72)	20 (28.16)	14 (17.28)	**0.025**
Diffused necrosis		0	2 (5.40)	4 (5.63)	7 (8.64)	0.075

*Type of diabetes*						
Type 2 diabetes mellitus	Physician and medical records	21 (95.45)	37 (100)	71 (100)	80 (98.76)	0.23
Type 1 diabetes mellitus		1 (4.54)	0 (0)	0 (1.7)	1 (1.23)	

*Antibiotic use*						
Yes	Questionnaire	0 (0)	4 (10.81)	57 (80.28)	79 (97.53)	**< 0.001**
No		22 (100)	33 (89.18)	14 (19.71)	2 (2.46)	

*Amputation*						
Yes	Questionnaire	6 (27.27)	8 (21.62)	13 (18.30)	2 (2.46)	**< 0.001**
No		10 (45.45)	26 (70.27)	30 (42.25)	71 (87.65)	
Topical debriding		6 (27.27)	3 (8.10)	28 (39.43)	8 (9.8)	

*Note:* The statistical analyses were conducted using the chi‐square and Fisher’s exact test, with the *R* statistical software (v.4.4). The statistical significance was considered as the *p* value less than 0.05. Bold values are statistically significant.

Abbreviation: NS: Nonsignificant.

**TABLE 4:** Bacterial species other than S. aureus in different wound grades.

**Bacterial species**	**Wound grade**					
	**Grade 1**	**Grade 2**	**Grade 3**	**Grade 4**	**Grade 5**	
*Edwardsiella*	0	0	0	0	1 (100) %	1 (100)
*Hafnia*	0	0	1 (100) %	0	0	1 (100)
*Enterobacter*	0	0	1 (50) %	0	1 (50) %	2 (100)
*Providencia*	1 (50) %	0	0	0	1 (50) %	2 (100)
*Citrobacter diversus*	2 (100) %	0	0	0	0	2 (100)
*Serratia marcescens*	0	0	1 (33.3) %	1 (33.3) %	1 (33.3) %	3 (100)
*Klebsiella oxytoca*	0	0	1 (25) %	2 (50) %	1 (25) %	4 (100)
*Citrobacter freundii*	1 (14.12) %	2 (28.5) %	0	2 (28.5) %	2 (28.5) %	7 (100)
*Klebsiella pneumoniae*	1 (10) %	2 (20) %	2 (20) %	1 (10) %	4 (40) %	10 (100)
*E. coli*	2 (5.4) %	6 (16.2) %	7 (18.9) %	11 (29.7) %	11 (29.7) %	37 (100)
*Proteus*	1 (50) %	0	0	1 (50) %	0	2 (100)
Total	8 (11.2) %	10 (14.3) %	13 (18.3) %	18 (25.3) %	22 (30.9) %	71 (100)

The present study also assessed various clinical and demographic characteristics of patients with DFUs. The results revealed that hypertension was the most common underlying disease (risk factor) among patients (*n* = 17). In addition, a significant proportion of patients (*n* = 15) suffered from cardiovascular diseases, renal failure (*n* = 9), and nine patients had neuropathy disease as well. Statistical analysis showed that the prevalence of these noted risk factors was not significant in DFU patients with MRSA or MSSA compared with DFU patients without MRSA or MSSA. This study also revealed that 69.5% of diabetic patients had a history of hospitalization in the past year. Moreover, the study evaluated the association of DFUs with other skin disorders, including deep soft tissue involvement, topical necrosis, cellulitis, bone involvement, and diffuse necrosis. According to the results, deep soft tissue involvement was the most prevalent skin disorder among patients (48.6%). Interestingly, the results showed that almost all patients with DFUs had Type 2 diabetes mellitus (*n* = 58), whereas only one patient had Type 1 diabetes mellitus. Antibiotic consumption was another factor evaluated in these patients; it was found that only 45% of patients had a history of antibiotic therapy. Furthermore, the study analysed the demographic characteristics of diabetic patients, including the history of amputation and topical debriding. According to the results, 14 patients reported amputation, while nine patients had a history of topical debriding (Table 3).

### 3.3. The Prevalence of Virulence Factors in *S. aureus* Isolates

**FIGURE 3:**
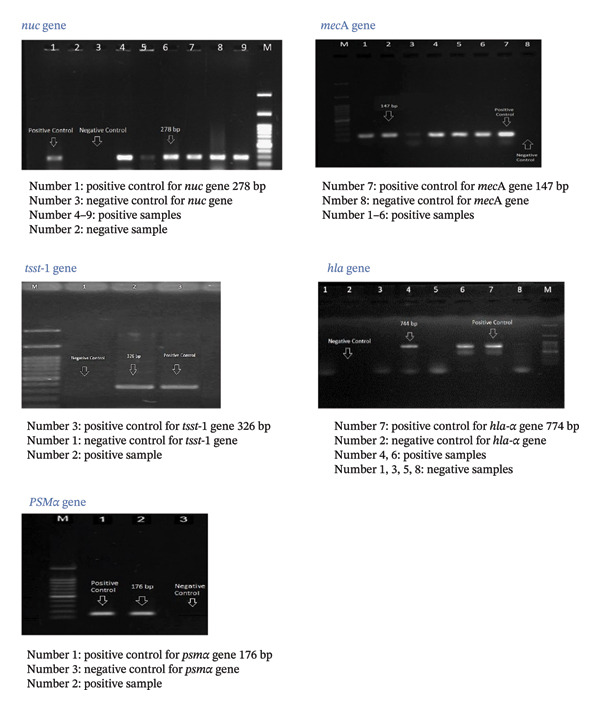
Representative of PCR images of *nuc* gene, *mecA* gene, *tsst-1* gene, *hla* gene, *PSMα* gene.

The findings revealed that the *hlα* and *psmα* genes were highly prevalent among both MSSA and MRSA isolates (Figure 3). The *hlα* gene was detected in 34 (57.6%) MRSA and nine (15.3%) MSSA isolates, whereas the *psmα* gene was found in 30 (50.8%) MRSA and 13 (22%) MSSA isolates. Interestingly, the *tsst* 1 gene was not prevalent among MSSA and MRSA isolates, and only nine isolates carried this gene (8.5% of MRSA isolates and 6.8% of MSSA isolates). Examining the presence of the *eta* and *etb* genes revealed that only 3.4% of MRSA and 1.7% of MSSA isolates carried the *eta* gene. Similarly, 5.1% of MRSA and 3.4% of MSSA isolates were *etb* positive. The results also demonstrated that *agr* Type I was the most prevalent *agr* type among *S. aureus* isolates (*n* = 16, 27.1% in MRSA and *n* = 11, 13.4% in MSSA), followed by *agr* Type II (*n* = 15, 25.4%). Also, *agr* Types III and IV were found in 8.4% (*n* = 5) and 13.55% (*n* = 8) of *S. aureus* isolates, respectively. These results are detailed in Table 5 and heatmaps A and B.

**TABLE 5:** Prevalence of virulence genes, *agr* types and biofilm formation power in MSSA and MRSA isolates.

Variables	MSSA *N* (%)	MRSA *N* (%)	*p* value
tsst1			
Yes	4 (6.8)	5 (8.5)	0.71
No	18 (30.5)	32 (54.2)	
hlα			
Yes	9 (15.3)	34 (57.6)	0.001
No	13 (22.0)	3 (5.1)	
psmα			
Yes	13 (22.0)	30 (50.8)	0.15
No	9 (15.3)	7 (11.9)	
agr			
No type	1 (1.7)	3 (5.1)	
1	11 (13.4)	16 (27.1)	
2	4 (6.8)	11 (18.6)	0.79
3	2 (3.4)	3 (5.1)	
4	4 (6.8)	4 (6.8)	
Eta			
Yes	1 (1.7)	2 (3.4)	0.99
No	21 (35.6)	35 (59.3)	
Etb			
Yes	2 (3.4)	3 (5.1)	0.99
No	20 (33.9)	34 (57.6)	
Biofilm			
No	3 (5.1)	7 (11.9)	
I	8 (13.6)	19 (32.2)	
S	7 (11.9)	5 (8.5)	0.45
W	4 (6.4)	6 (10.2)	
SCC*mec* I		0	
SCC*mec* II		6 (3.61)	
SCC*mec* III		18 (10.84)	
SCC*mec* Iva		4 (2.40)	
SCC*mec* Ivb		4 (2.40)	
SCC*mec* Ivc		4 (2.40)	
SCC*mec* V		1 (0.60)	
N		22 (36.52)	

*Note:* I, intermediate; S, strong; W, weak.

**FIGURE 4:**
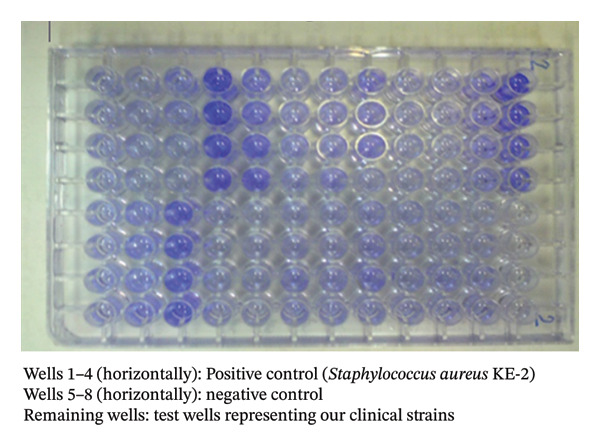
Biofilm formation assay. Wells 1–4 (horizontally): positive control (*Staphylococcus aureus* KE‐2), wells 5–8 (horizontally): negative control, the remaining wells: test wells representing clinical isolates.

This results of this study indicated that among the 59 *S. aureus* isolates analysed, most of the isolates (*n* = 27) produced intermediate biofilm, while 12 isolates displayed strong biofilm, and 10 isolates produced weak biofilm (Figure 4). Interestingly, 10 *S. aureus* isolates were unable to form biofilm. The heatmaps in Figure 2 illustrate these findings. Furthermore, the findings showed that out of 59 *S. aureus* isolates tested, 43 isolates were positive for haemolysin *α*, but only 27 isolates were confirmed to be haemolysin *α* positive based on phenotypic test results. Among the remaining isolates, 11 isolates were positive for haemolysin *β*, 15 isolates were positive for haemolysin *γ*, and six isolates were positive for both haemolysin *α* and *β*.

In the present study, all *S. aureus* isolates were subjected to genotypic detection of the *mec*A gene. The results revealed that 37 out of 59 *S. aureus* isolates were positive for *mec*A and therefore identified as MRSA, while the remaining 22 MSSA isolates were negative for *mec*A, and these findings were consistent with phenotypic test results using a cefoxitin disk. To detect the type of *mec*A gene, SCC*mec* typing was performed using multiplex PCR. The findings showed that SCC*mec* Type III was the most common subtype, which was present in 18 isolates (48.6%), followed by SCC*mec* Type II, which was present in six isolates (16.2%). Additionally, SCC*mec* Type IVa, SCC*mec* Type IVb and SCC*mec* Type IVc were each detected in four isolates, while SCC*mec* Type V was found in only one isolate (Table 5).

### 3.4. Antimicrobial Resistance Pattern of *S. aureus* Isolates

**FIGURE 5:**
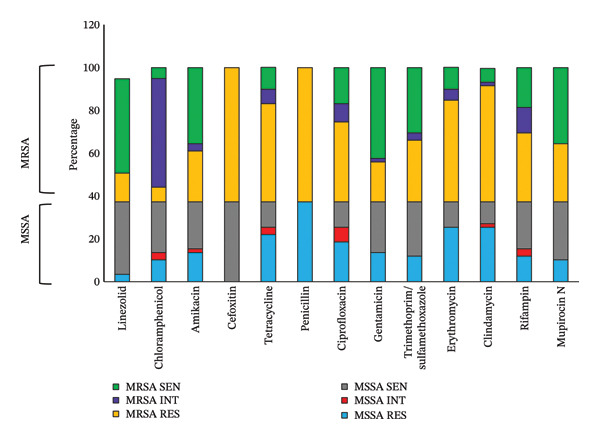
Antibiotic susceptibility pattern of diabetic foot infections due to *S. aureus*. MSSA RES: methicillin‐susceptible *Staphylococcus aureus* resistant, MSSA INT: methicillin‐susceptible *Staphylococcus aureus* intermediate, MSSA SEN: methicillin‐sensitive *Staphylococcus aureus* susceptible, MRSA RES: methicillin‐resistant *Staphylococcus aureus* resistant, MRSA INT: methicillin‐resistant *Staphylococcus aureus* intermediate, MRSA SEN: methicillin‐resistant *Staphylococcus aureus* susceptible.

The antibiogram results of all 59 *S. aureus* isolates are presented in Figure 5. It was observed that all MRSA isolates (*n* = 37) were resistant to cefoxitin (62.7%) and penicillin (62.7%). In addition, MRSA isolates showed high resistance to clindamycin (54.2%), erythromycin (47.5%) and tetracycline (45.8%) and high sensitivity to linezolid (44%), gentamicin (42.4%), amikacin (35.6%) and mupirocin (35.6%), respectively. Besides, MSSA isolates showed high resistance to penicillin (37.3%), clindamycin (25.4%) and erythromycin (25.4%) and high sensitivity to cefoxitin (37.3%), linezolid (33.9%) and mupirocin (27.1%).

## 4. Discussion

Numerous scientific studies have demonstrated that DFIs are often caused by multiple microorganisms, with *S. aureus* being the most frequently isolated pathogen [35, 50, 51]. In this study, 211 clinical samples were collected from patients with DFUs, and *S. aureus* was the most prevalent pathogen, accounting for 59 isolates, followed by *E. coli* (*n* = 37) and *K. pneumoniae* (*n* = 10). These findings are consistent with the results of a meta‐analysis by Macdonald et al., who analysed 16,159 patients included in 112 studies and found that *S. aureus* was the most commonly identified isolate, followed by *Pseudomonas* spp., *E. coli* and *Enterococcus* [52].

The aetiology of DFIs has been widely studied, and Gram‐positive bacteria have been identified as the predominant causative agents [53]. This finding has been supported by numerous studies conducted in Iran [54–56] and developed countries such as North America and Europe [57, 58]. In a large multi‐institutional study in the USA, 77% of DFIs were caused by Gram‐positive aerobes, while Gram‐negative aerobes accounted for 21.2% of cases [59]. However, some studies have reported a higher prevalence of Gram‐negative bacteria in DFIs [60, 61]. In a study conducted by Raja, Gram‐negative bacteria accounted for 52% of the isolates collected from 194 patients with DFUs. Among Gram‐negative bacteria, *Proteus* was found to be the most common pathogen (28%), while *S. aureus* was predominant among Gram‐positive bacteria (44%) [62]. In another study by Hatipoglu et al., both aerobic Gram‐positive and Gram‐negative organisms were isolated from DFIs with almost equal frequency (48.4% vs. 48.4%) during 1989–2011 [63]. However, differences in the prevalence of Gram‐positive and Gram‐negative organisms may be attributed to variations in geographic areas and healthcare systems of different countries, in treating and managing DFIs [64]. In the present study, 76.2% of the patients were male and over 62 years old, and this observation may reflect the role of different lifestyles and occupations in DFI incidence [65, 66]. Additionally, the prevalence of MRSA and MSSA isolates was similar in women, but in men, the prevalence of MRSA isolates was twice that of MSSA isolates. This finding is consistent with the results of previous studies, showing that male gender is significantly associated with an increased risk of MRSA infection [67]. Ahmadi et al. showed that poor attention to personal hygiene in men could contribute to higher prevalence of MRSA infection in this group [68]. However, MRSA strains were highly prevalent in both genders, indicating a need for effective infection control measures. Moreover, a number of studies have indicated the presence of toxicogenic *Staphylococcus aureus* strains (harbouring exfoliation‐, PVL‐ or TSST‐encoding genes) in DFI, particularly in Grade 4, with systemic impact [18]. As demonstrated by Sotto et al. [50], isolates from higher grade (≥ 2) ulcers exhibited a significantly higher propensity to carry the *sea, sei, lukE and hlgv* genes, thereby distinguishing infected from colonized wounds. Furthermore, a recent meta‐analysis revealed that major toxin‐encoding and haemolysin genes (e.g., hla, hlg, hlgV) are highly prevalent in *S. aureus* from DFI, suggesting that a virulence gene profile correlates with ulcer severity and may aid in predicting poor outcomes [69]. Furthermore, as indicated by the findings of preceding studies, there is a demonstrable correlation between elevated Wagner grades and an augmentation in antimicrobial resistance among *Staphylococcus aureus* isolates derived from DFUs. As demonstrated in the study by Xie et al., *S. aureus* isolates from higher Wagner grades exhibited a greater degree of resistance to penicillin, cefoxitin, erythromycin, clindamycin, tetracycline and ciprofloxacin [70]. Similarly, the report by Atlaw et al. demonstrated that more advanced ulcers were associated with reduced susceptibility to gentamicin and amikacin, as well as increased resistance to trimethoprim–sulfamethoxazole. These findings are consistent with the results of the present study, which demonstrate a higher frequency of MRSA in ulcers classified as Wagner grade ≥ 3, emphasizing the significance of early diagnosis and appropriate wound management [71].

The current study results showed that 62.7% of *S. aureus* strains isolated from DFIs were MRSA. This finding is consistent with the results of two meta‐analyses conducted on Iranian populations, which found that the prevalence of MRSA in DFIs was between 52.7% and 55% [69, 72]. It is worth noting that the prevalence of MRSA strains in DFIs varies from country to country, with higher rates observed in less developed countries (between 66% and 68%) [73]. In a cohort study conducted by Mariam et al., 169 out of 279 participants (60.6%) had Type 2 diabetes mellitus, while in the present study, this figure was approximately 98%. Additionally, the prevalence of hypertension in Mariam et al.’s study was 71.4%, whereas in the present study, about 29% of diabetic patients had high blood pressure [74]. Interestingly, in both studies, hypertension was the most common underlying disease in patients with DFUs. In the current investigation, the antibiotics that exhibited the highest efficacy against *S. aureus* infection were gentamicin and mupirocin, with corresponding sensitivity rates of 66.1%, 66.1% and 62.7%, respectively. However, Sanchez et al. identified levofloxacin as the most potent antibiotic against Gram‐positive bacteria [75], while penicillin was deemed the least effective antibiotic, which is consistent with the present study result. Perim et al. demonstrated that imipenem was the most potent antibiotic against both Gram‐positive and Gram‐negative bacteria in DFUs [76]. In a 2012 systematic review on the treatment of DFIs, 12 studies met the criteria for comparing different antibiotic regimens to treat soft tissue infections (including MRSA). Nevertheless, none of the studies provided clear evidence of the superiority of one regimen over another based on route of administration or duration of antibiotic therapy [77]. One of the objectives of this study was to investigate the prevalence of virulence genes (such as *hla*, *psma*, *agr*, *eta*, *etb* and *tsst*‐1) and SCC*mec* types in MRSA isolates. The ability of MRSA to lyse red blood cells is due to the expression of different types of haemolysins, namely *α*, *β*, *δ* and *γ*‐haemolysins. The *hla* gene encodes alpha‐haemolysin, a pore‐forming toxin [78]. The expression of *hla* is regulated by a complex regulatory network. It has been observed that *hla* is upregulated during infection [79]. The prevalence of the *hla* gene in the present study was 57.6%, and a significant correlation was observed between the presence of *hla* and MRSA clinical isolates (*p* value < 0.05). These findings are consistent with those previously reported by Rossato et al., indicating that the *hla* gene was present in 87.6% of MRSA isolates obtained from hospitals in Brazil [80]. PSMs (phenol‐soluble modulins) are membrane‐damaging toxins produced by most *S. aureus* strains. They cause the lysis of human neutrophils, monocytes, erythrocytes and osteoblasts, thereby increasing tissue toxicity [81]. The results showed that this toxin was the second most prevalent toxin in MRSA (50.8%) and MSSA (22%) isolates. TSST‐1 is a superantigen produced by *S. aureus*, which is responsible for the development of toxic shock syndrome (TSS) [82].

Several studies have reported the prevalence of the *tsst* gene in *S. aureus* strains isolated from DFUs, ranging from 8% to 13% [83, 84]. However, studies conducted in Algeria [85] and Africa [86] have reported no *S. aureus* strains positive for the *tsst* gene. The present study results revealed the low prevalence of the *tsst* gene in both MRSA (8.5%) and MSSA (6.8%) isolates, which is in contrast to another study in Iran, reporting a high prevalence of the *tsst-1* gene in both MRSA (69.8%) and MSSA (56.0%) isolates [87]. Moreover, exfoliative toxins, such as *eta* and *etb*, are serine proteases secreted by *S. aureus*, which facilitate bacterial invasion of the skin [88]. The findings showed that the prevalence of the *eta* gene was 1.7% and 3.4% in MSSA and MRSA isolates, respectively, while the prevalence of *etb* was 3.4% in both MSSA and MRSA isolates.

It is widely recognized that *agr* types play a crucial role in regulating virulence factors and are frequently associated with isolates harbouring significant virulence factors. This study revealed that the most common *agr* type was *agr* Type I (27.1%), followed by *agr* Type II (18.6%), which is consistent with previous reports in the literature [89–91]. In MRSA isolates, SCC*mec* III was the most prevalent subtype, accounting for 48.6% of the isolates, while SCC*mec* II was present in 16.2% of the isolates. A study conducted in China found that all 40 MRSA strains harboured SCC*mec* III [92]. Similarly, a recent study by Rahimi et al. reported that SCC*mec* III was detected in 86.3% of MRSA isolates collected from a reference hospital in Iran [67]. In contrast, a recent study in China showed that SCC*mec* II was the predominant subtype harboured by 77.8% of MRSA isolates from DFIs [93]. Moreover, Kananizade et al. in Iran demonstrated that the most prevalent SCC*mec* type was Type IV (46.7%), followed by Type III (30.0%), and discussed the entry and increase of this subtype in clinical settings [94]. However, the results of the present and other recent similar studies in Iran do not currently confirm their findings. In addition, in the present study, the antibiotic susceptibility patterns of SCC*mec* Type III–positive isolates were found to be comparable to those of isolates harbouring SCC*mec* Types II, IV and V.

## 5. Conclusion

The present study revealed that the microbial composition of DFUs in patients was in line with prior reports, while their antimicrobial profile exhibited minor variations. These findings emphasize the critical importance of a comprehensive understanding of patients’ medical records and underlying diseases; in addition, accurate evaluation of microbial prevalence and antibiotic susceptibility is necessary to effectively manage DFIs and prevent amputation, prolonged hospitalization and infection‐related morbidity.

### 5.1. Limitations

With great sorrow, our colleague (Professor Shahin Najar‐Peerayeh) passed away in the middle of the study. She was a brilliant and compassionate researcher who fought against COVID‐19 and could not continue her research journey. Peace be upon her.

**Nomenclature**
*S. aureus*
: *Staphylococcus aureus*
MRSA: Methicillin‐resistant *S. aureus*
DFUs: Diabetic foot ulcers
ASTs: Antibiotic susceptibility tests
MSSA: Methicillin‐susceptible *S. aureus*
*Agr*
: Accessory gene regulator
*Hlα*
: α‐haemolysin
*Psmα*
: Phenol‐soluble modulins
*ETA*
: Exfoliative Toxin A
*ETB*
: Exfoliative Toxin B
*TSST*‐*1*
: Toxic shock syndrome toxin‐1
*SCCmec*
: Staphylococcal cassette chromosome *mec*
VRSA: Vancomycin‐resistant *S. aureus*

## Author Contributions

Bita Bakhshi and Shahin Najar‐Peerayeh designed, conceptualized and supervised the work and designed the experiments; Haniyeh Khalili and Mona Mahrooghi conducted the experiments, collected data and wrote the first version of the manuscript; Bita Bakhshi, Hossein Najd Sepas and Shabnam Zeighamy Alamdary analysed the data and reviewed the manuscript.

## Funding

This study was not funded.

## Disclosure

All authors read and approved the final version of the manuscript.

## Ethics Statement

This project was approved by the Ethics Committee of Tarbiat Modares University of Medical Sciences. Informed written consent was taken from each participating patient or their parent prior to the study.

## Consent

Please see the Ethics Statement.

## Conflicts of Interest

The authors declare no conflicts of interest.

## Data Availability

The datasets used and/or analysed during the current study are available from the corresponding author with no restriction.
